# Hypertonic saline infusion for treating intracranial hypertension after severe traumatic brain injury

**DOI:** 10.1186/s13054-018-1963-7

**Published:** 2018-02-20

**Authors:** Halinder S. Mangat

**Affiliations:** Department of Neurology & Neurological Surgery, Cornell University Joan and Sanford I Weill Medical College, 525 East 68 street, F-610, New York, NY 10065 USA

**Keywords:** Traumatic brain injury, Hyperosmolar, Hypertonic saline, Intracranial hypertension, Intracranial pressure

## Abstract

Traumatic brain injury (TBI) remains a major cause of mortality and disability. Post-traumatic intracranial hypertension (ICH) further complicates the care of patients. Hyperosmolar agents are recommended for the treatment of ICH, but no consensus or high-level data exist on the use of any particular agent or the route of administration. The two agents used commonly are hypertonic saline (HTS) and mannitol given as bolus therapy. Smaller studies suggest that HTS may be a superior agent in reducing the ICH burden, but neither agent has been shown to improve mortality or functional outcome. In a recently published analysis of pooled data from three prospective clinical trials, continuous infusion of HTS correlated with serum hypernatremia and reduced ICH burden in addition to improving 90-day mortality and functional outcome. This lays the foundation for the upcoming continuous hyperosmolar therapy for traumatic brain-injured patients (COBI) randomized controlled trial to study the outcome benefit of continuous HTS infusion to treat ICH after severe TBI. This is much anticipated and will be a high impact trial should the results be replicated. However, this would still leave a question over the use of mannitol bolus therapy which will need to be studied.

Traumatic brain injury (TBI) is one of the major causes of death and disability, and contributes to 30% of all injury-related mortality. After severe TBI, prompt surgical and medical management of patients reduces mortality. Guidelines for the management of severe TBI have been formulated and published for the past two decades by the Brain Trauma Foundation (BTF), and adherence to these has resulted in a significant reduction in mortality [1, 2].

The primary interventions that have maximal impact and have led to the several-fold reduction in mortality from severe TBI over the past decades are immediate surgical intervention and subsequent care by specialist intensivists. In a majority of patients who have undergone craniotomy for surgical evacuation of an extra-axial clot or rarely decompressive craniectomy for severe swelling, as well as in patients who may not have a surgical lesion, post-traumatic intracranial hypertension (ICH) poses a threat to life and quality of life after survival. There are few proven therapies for the efficient treatment of ICH, yet there is a paucity of prospective randomized clinical trials for those agents.

Hypertonic solutions such as mannitol and hypertonic saline (HTS) are recommended early in the management of ICH after severe TBI [1]. They provide therapeutic benefit along with a wide therapeutic margin. The most recent BTF guidelines stated “although hyperosmolar therapy may lower intracranial pressure, there was insufficient evidence about effects on clinical outcomes to support a specific recommendation, or to support use of any specific hyperosmolar agent”. The current recommendation for the use of mannitol to treat ICH is carried from the previous edition of the guidelines “to maintain sufficient recognition of the potential need for hyperosmolar therapy to reduce intracranial pressure, while acknowledging that more research is needed to inform more specific recommendations” [1]. While mannitol has been the traditional agent of choice supported by older studies, the use of HTS is increasing and is supported by several recent studies, albeit small or heterogeneous ones [3–8]. Reduction in intracranial pressure (ICP) has been consistently demonstrated with both mannitol and HTS, but there is a suggestion that HTS provides a more robust and durable effect in lowering ICP [3, 7]. Recent research has now focused on establishing superiority between HTS and mannitol administered as bolus therapy.

There have also been a few small prospective single-arm studies involving patients with different intracranial pathologies that have shown that the use of continuous HTS infusion to achieve hypernatremia is beneficial in reducing ICP [9, 10]. Asehnoune et al. analyzed pooled data from three prospective clinical trials involving TBI patients, and compared data from one center that used continuous HTS infusion as first-line ICH therapy to other centers that administered HTS as bolus therapy only, as part of a systematic, guidelines-based tiered therapy for raised ICP after severe TBI [11]. With propensity score analysis adjusted for defined confounders of outcomes, the adjusted hazards ratio for survival and 90-day functional outcome with continuous HTS therapy were significantly greater than that for intermittent therapy. No significant adverse effects such as renal failure and neurological complications were seen. In pediatric patients treated with HTS, sustained hypernatremia has been shown to be associated with thrombocytopenia, renal failure, neutropenia, and acute respiratory distress syndrome, but these have not been observed extensively in adults, though associated hyperchloremia may be associated with increased mortality [12–15].

The above study is the first large prospective dataset to demonstrate survival benefit with use of HTS, and provides a foundation for the planned COBI trial (continuous hyperosmolar therapy for traumatic brain-injured patients; NCT03143751), which has been approved and funded to commence shortly [16]. COBI will study the use of continuous HTS infusion for a minimum of 48 h in moderate to severe TBI patients aged 18–80 years using the primary outcome measure of Glasgow Outcome Score–extended (GOS-E).

While this trial will answer the question of utility of continuous HTS in improving outcomes, the question of the efficacy of HTS in comparison to mannitol bolus therapy, which is currently the preferred treatment, will remain unanswered. The mode of administration is important since bolus therapy reduces elevated ICP immediately by improving cerebral hemodynamics, but it is a reactive measure. After repeated doses over several hours, HTS and mannitol cause a reduction in brain water and further reduction in ICP. In comparison, continuous infusion is associated with hypernatremia and hyperosmolality, which gradually cause cerebral dehydration and pre-emptively reduce ICP.

And what of mannitol; will it be abandoned if the results of the COBI trial confirm the current findings? That may not happen so readily, with the primary reason being the ease of peripheral intravenous administration versus the need for central venous catheters that are required for administering hypertonic saline. It may be that mannitol remains the hyperosmolar agent of choice for early resuscitation, while HTS may be adopted for maintenance therapy, even though data for that also favors HTS [5].

## Conclusion

Hyperosmolar therapy offers a means to reduce ICH after severe TBI. There are no high-level data on the superiority of mannitol versus HTS in reducing ICH burden or improving outcomes. New data suggest that continuous infusion of HTS reduces ICH burden and improves survival and functional outcomes. The COBI trial will study the outcome benefit of continuous HTS infusion therapy in moderate and severe TBI patients, and may well be a hallmark trial for the effect of hyperosmolar agents in not only reducing ICH but also improving survival and functional outcomes.

## Abbreviations

BTF: Brain Trauma Foundation
COBI: Continuous hyperosmolar therapy for traumatic brain-injured patients
GOS-E: Glasgow Outcome Score–Extended
HTS: Hypertonic saline
ICH: Intracranial hypertension
ICP: Intracranial pressure
TBI: Traumatic brain injury

## References

[1] Carney N, Totten AM, O'Reilly C, Ullman JS, Hawryluk GW, Bell MJ, Bratton SL, Chesnut R, Harris OA, Kissoon N (2017). Guidelines for the management of severe traumatic brain injury, fourth edition. Neurosurgery..

[2] Gerber LM, Chiu YL, Carney N, Hartl R, Ghajar J (2013). Marked reduction in mortality in patients with severe traumatic brain injury. J Neurosurg..

[3] Mangat HS, Chiu Y-L, Gerber LM, Alimi M, Ghajar J, Härtl R (2015). Hypertonic saline reduces cumulative and daily intracranial pressure burdens after severe traumatic brain injury. J Neurosurg..

[4] Roquilly A, Mahe PJ, Latte DD, Loutrel O, Champin P, Di Falco C, Courbe A, Buffenoir K, Hamel O, Lejus C (2011). Continuous controlled-infusion of hypertonic saline solution in traumatic brain-injured patients: a 9-year retrospective study. Crit Care..

[5] Koenig MA, Bryan M, Lewin JL, Mirski MA, Geocadin RG, Stevens RD (2008). Reversal of transtentorial herniation with hypertonic saline. Neurology..

[6] Cottenceau V, Masson F, Mahamid E, Petit L, Shik V, Sztark F, Zaaroor M, Soustiel JF (2011). Comparison of effects of equiosmolar doses of mannitol and hypertonic saline on cerebral blood flow and metabolism in traumatic brain injury. J Neurotrauma..

[7] Ichai C, Armando G, Orban JC, Berthier F, Rami L, Samat-Long C, Grimaud D, Leverve X (2009). Sodium lactate versus mannitol in the treatment of intracranial hypertensive episodes in severe traumatic brain-injured patients. Intensive Care Med..

[8] Eisenberg HM, Frankowski RF, Contant CF, Marshall LF, Walker MD (1988). High-dose barbiturate control of elevated intracranial pressure in patients with severe head injury. J Neurosurg..

[9] Qureshi AI, Suarez JI, Bhardwaj A, Mirski M, Schnitzer MS, Hanley DF, Ulatowski JA (1998). Use of hypertonic (3%) saline/acetate infusion in the treatment of cerebral edema: effect on intracranial pressure and lateral displacement of the brain. Crit Care Med..

[10] Hauer EM, Stark D, Staykov D, Steigleder T, Schwab S, Bardutzky J (2011). Early continuous hypertonic saline infusion in patients with severe cerebrovascular disease. Crit Care Med..

[11] Asehnoune K, Lasocki S, Seguin P, Geeraerts T, Perrigault PF, Dahyot-Fizelier C, Paugam Burtz C, Cook F, Demeure Dit Latte D, Cinotti R (2017). Association between continuous hyperosmolar therapy and survival in patients with traumatic brain injury—a multicentre prospective cohort study and systematic review. Crit Care.

[12] Gonda DD, Meltzer HS, Crawford JR, Hilfiker ML, Shellington DK, Peterson BM, Levy ML (2013). Complications associated with prolonged hypertonic saline therapy in children with elevated intracranial pressure. Pediatr Crit Care Med..

[13] Froelich M, Ni Q, Wess C, Ougorets I, Hartl R (2009). Continuous hypertonic saline therapy and the occurrence of complications in neurocritically ill patients. Crit Care Med..

[14] Riha HM, Erdman MJ, Vandigo JE, Kimmons LA, Goyal N, Davidson KE, Pandhi A, Jones GM (2017). Impact of moderate hyperchloremia on clinical outcomes in intracerebral hemorrhage patients treated with continuous infusion hypertonic saline: a pilot study. Crit Care Med..

[15] Tan SK, Kolmodin L, Sekhon MS, Qiao L, Zou J, Henderson WR, Griesdale DE (2016). The effect of continuous hypertonic saline infusion and hypernatremia on mortality in patients with severe traumatic brain injury: a retrospective cohort study. Can J Anaesth..

[16] Roquilly A, Lasocki S, Moyer JD, Huet O, Perrigault PF, Dahyot-Fizelier C, Seguin P, Sharshar T, Geeraerts T, Remerand F (2017). COBI (COntinuous hyperosmolar therapy for traumatic Brain-Injured patients) trial protocol: a multicentre randomised open-label trial with blinded adjudication of primary outcome. BMJ Open..

